# Over-production of exopolysaccharide by *Lacticaseibacillus rhamnosus* CNCM I-3690 strain cutbacks its beneficial effect on the host

**DOI:** 10.1038/s41598-023-32116-3

**Published:** 2023-04-14

**Authors:** R. Martín, A. Benítez-Cabello, S. Kulakauskas, M. V. C. Viana, C. Chamignon, P. Courtin, C. Carbonne, F. Chain, H. P. Pham, Muriel Derrien, L. G. Bermúdez-Humarán, M. P. Chapot-Chartier, T. Smokvina, P. Langella

**Affiliations:** 1 Commensal and Probiotics-Host Interactions Laboratory, INRAE, AgroParisTech, Micalis Institute, Université Paris-Saclay, 78350 Jouy-en-Josas, France; 2 Dynamics of Bacterial Cell Wall Laboratory, INRAE, AgroParisTech, Micalis Institute, Université Paris-Saclay, 78350 Jouy-en-Josas, France; 3 Laboratory of Cellular and Molecular Genetics, Federal University of Minas Gerais, Belo Horizonte, Brazil; 4 Parean Biotechnologies, 35400 Saint-Malo, France; 5 Danone Nutricia Research, Palaiseau, France

**Keywords:** Microbiology, Applied microbiology, Bacteria, Bacteriology

## Abstract

Most lactobacilli produce extracellular polysaccharides that are considered to contribute to the probiotic effect of many strains. *Lacticaseibacillus rhamnosus* CNCM I-3690 is an anti-inflammatory strain able to counterbalance gut barrier dysfunction. In this study ten spontaneous variants of CNCM I-3690 with different EPS-production were generated and characterized by their ropy phenotype, the quantification of the secreted EPS and genetic analysis. Amongst them, two were further analysed in vitro and in vivo: an EPS over-producer (7292) and a low-producer derivative of 7292 (7358, with similar EPS levels than the wild type (WT) strain). Our results showed that 7292 does not have anti-inflammatory profile in vitro, and lost the capacity to adhere to the colonic epithelial cells as well as the protective effect on the permeability. Finally, 7292 lost the protective effects of the WT strain in a murine model of gut dysfunction. Notably, strain 7292 was unable to stimulate goblet cell mucus production and colonic IL-10 production, all key features for the beneficial effect of the WT strain. Furthermore, transcriptome analysis of colonic samples from 7292-treated mice showed a down-regulation of anti-inflammatory genes. Altogether, our results point out that the increase of EPS production in CNCM I-3690 impairs its protective effects and highlight the importance of the correct EPS synthesis for the beneficial effects of this strain.

## Introduction

The genus *Lactobacillus,* recently reclassified into 25 new genera^1^, including the emended genus *Lactobacillus,* comprises 261 species (in June 2021). They present an adaptability that has allowed them to colonize a large number of ecosystems, including the human body and within the gastrointestinal (GIT) and vaginal tracts in humans. The probiotic potential of certain strains belonging to this genus has been widely studied. In this sense, the ability of *Lacticaseibacillus rhamnosus* to maintain gut homeostasis^2^ and restore dysbiosis-mediated diseases has been demonstrated previously^3^. Within this species, *L. rhamnosus* GG (LGG) is one of the most studied. Thus, several beneficial effects have been associated in clinical trials and human intervention studies, as well as its ability to adhere to the intestinal epithelial layer.

The interaction of lactobacilli with the host GIT is mediated by the molecules that make up bacterial surface^4^, which in turn determines the adherence to the host tissue, the interaction with other microorganisms, and, ultimately, their survival. The interaction of these structures and, consequently, the effect produced by the bacteria can be reduced or enhanced by the presence of extracellular polysaccharides (PS) surrounding the cell. These PS can either remain attached to the bacterial surface after their synthesis and may form a capsule or they can be released into the surrounding medium in the form of exopolysaccharides (EPS), resulting in a ropy phenotype. PS are polymeric carbohydrate molecules, composed of a series of monosaccharides, connected to each other by glycosidic bonds. According to their chemical composition, PS are divided into homopolysaccharides (HoPS) and heteropolysaccharides (HePS)^5,6^. There is a great diversity among the lactic acid bacteria (LAB) as HePS could vary in composition, structure, molecular mass, and production^5,7,8^. All these characteristics strongly contribute to the specific PS characteristics, such as solubility, rheological properties, and physicochemical and biological functionality.

Unlike other PS, which are covalently attached to the cell wall, EPS are secreted into the growth medium or loosely attached to the cell surface^9^. The diversity of bacterial EPS structures is vast. Since the properties of EPS depend on their structure, the spectrum of technological and functional properties of EPS is very broad. EPS produced by LAB contribute to the textural properties of dairy products. EPS have also been suggested to influence bacteria-host interactions, especially by affecting host cell and mucosa adhesion, or bacterial-mediated immunomodulation^9^. EPS from *lactobacilli* have been reported to have a large number of pharmacological applications, such as immunomodulatory, blood cholesterol-reducers, nutraceuticals, anti-allergic, etc.^10–12^.

EPS produced by probiotic bacteria have been reported to control the inflammatory mechanism of immune cells^13^, increase colonisation in the gut^10^ and modulate the intestinal microbiota^14^. Indeed, EPS may serve as source of nutrients for the resident microbiota. Previous studies on either purified EPS^14–16^ or EPS-producing and non-producing strains^17^ showed the ability of EPS to mediate microbe–microbe interactions. However, the contribution of the EPS overproduction by *Lactobacillus* strains in relation to host effects has been largely underexplored.

Probiotic strains may represent in the future potential strategies for the treatment and prevention of gut dysfunction, with a significant impact on human health. This is the case of *L. rhamnosus* CNCM-I 3690, first described by Grompone et al.^18^ as an excellent protector of oxidative stress through an anti-inflammatory effect, and subsequently as a notable protector of the intestinal barrier functions in a murine model of inflammation^19^. This protection was then associated with maintaining modulated globular cells and mucus layer modulated and countering changes in local and systemic lymphocytes^4^. Furthermore*, L. rhamnosus* CNCM I-3690 has 7 putative surface adhesins and among them, SpaFED pili has been previously found to be key in its beneficial effects on the host^4^.

In the present study, we analysed the contribution of EPS production to the anti-inflammatory properties of *L. rhamnosus* CNCM I-3690. We selected the high EPS-producer and its lower producer derivate and analysed in a murine mode of low-grade inflammation in vivo, for both host and gut microbiota interactions.

## Material and methods

### Bacterial strains, cell lines, and culture conditions

*L. rhamnosus* CNCM I-3690 and its variants were grown in Man, Rogosa, and Sharpe (MRS) medium (Difco, USA) at 37 °C.

Human Caco-2 (ATCC, Port Down, UK), HT-29 (HTB-38, LGC Standards), and HT-29 MTX cell lines were used to perform the assays according to standard protocols. Caco-2 cells were grown in Dulbecco’s modified Eagle’s minimum essential medium (DMEM, pH 7.4) (Invitrogen, Waltham, MA USA) supplemented with 10% inactivated fetal bovine serum (FBS) (Lonza, Levallois-Perret, France), 25 mM glucose, 1% penicillin/streptomycin (PS), 1% glutamine (Invitrogen) and 1% non-essential amino acid solution (Invitrogen). HT-29 and HT-29 MTX were cultured under the same conditions, but without non-essential amino acids.

### Isolation of *L. rhamnosus* CNCM I-3690 variants affected in EPS production

Variants with altered EPS production were isolated using Todd Hewitt broth with 0.5% yeast extract with a low agar concentration (0.03%)^20,21^. The extent of free displacement of bacteria in this medium depends on the viscosity of the semi-liquid (SL) medium and EPS production. Consequently, the EPS-producing variants were isolated as slower sedimenting derivatives; EPS-negative variants as faster sedimenting derivatives.

### Quantification of EPS production

To quantify EPS production, bacteria were grown on Durapore membrane filters (0.22 µm GV, Millipore) placed on MRS agar plates. Bacteria were harvested and resuspended in MilliQ H_2_O. The bacterial suspension was adjusted to 50 OD in 500 µl, heated for 20 min at 100 °C to inactivate potential enzymes able to degrade polysaccharides and then centrifuged for 20 min at 20,000 g. The supernatant was recovered and EPS were precipitated by ethanol as previously described^22^. The total neutral sugar amount in the EPS extracts (expressed in glucose equivalent) was quantified with glucose as standard^23^.

### Genomic DNA extraction

Genomic DNA was extracted from 5 mL of an overnight culture with a first step of enzymatic lysis with the following cocktail: mutanolysin (233.3 µ/mL, Sigma); lysostaphin (13.3 µ/mL, Sigma) and lysozyme (50 mg/mL, Sigma) followed by incubations with RNAse A (10 mg/mL; Thermofisher) and proteinase K (50 mg/mL, Euromedex). DNA purification was performed with DNA extraction kit (Genomic DNA Buffer Set and Genomic Tips, Qiagen) according to manufacturer’s instructions. DNA was re-suspended in TE buffer and concentration was measured with NanoDrop (NanoDrop 1000, ThermoScientific).

### Genome analyses

The genomes were sequenced by Eurofins, using NanoPore GridION platform with Flow Cells version R9.4.1 (Oxford Nanopore Technologies, UK) and assembled using the softwares Flye (https://github.com/fenderglass/Flye) and Medaka (https://github.com/nanoporetech/medaka). The presence of plasmids was verified using PlasmidFinder (https://cge.food.dtu.dk/services/PlasmidFinder/). The genomes were annotated using RAST-Tk from BV-BRC (https://www.bv-brc.org/app/Annotation) and EggNOG-mapper (http://eggnog-mapper.embl.de). Variations between the reference genome WT and the variants 7259, 7292 and 7358 were identified using two approaches. Large variations were verified using Artemis Comparison Tool v18.1.0 (http://sanger-pathogens.github.io/Artemis/ACT/). SNPs were identified using the tool Variation Analysis from BV-BRC (https://www.bv-brc.org/app/Variation), keeping variations with ≥ 90% coverage. Gene sequences were aligned and visualized using Jalview v2.11.2.5 (https://www.jalview.org/), with MUSCLE v3.8.31 alignment algorithm^24^.

### In vitro immuno-modulatory, permeability, and adhesion tests

The anti-inflammatory properties of the *L. rhamnosus* derivatives were determined by measuring their capacity to suppress the secretion of the pro-inflammatory IL-8 in TNF-α stimulated HT-29 cells. Briefly, suspensions of 1 × 10^5^ differentiated HT-29 cells were seeded in 24 wells plates in the medium described above and incubated for 7 days at 37 °C in 10% CO_2_ air atmosphere until confluence (~ 1.83 × 10^6^ cells/well). The medium was changed daily. Twenty-four hours before co-culture, the serum concentration in the medium was reduced to 5%. Before co-culture, the medium was replaced with fresh medium supplemented with 5% SFV and 5 ng/mL of recombinant human TNF-α (Preprotech, USA) to stimulate the IL-8 production. Bacterial suspensions were added at a multiplicity of infection (MOI) of 1:40.

For the permeability assay, the Caco-2 cell line was co-cultured with the bacterial strains. Briefly, a suspension of 1 × 10^5^ Caco-2 cells was plated on Transwell semi-permeable filter supports (12 mm diameter wells, polystyrene membranes with 0.4 µm pores, Costar, Corning) with Trans-Epithelial Electrical Resistance (TEER) readings > 900 ohms cm^−2^. Then, TEER was then measured and 1 × 10^6^ CFU of the bacterial suspension per well was added onto the apical surface. Three hours later, cells were stimulated by adding 100 ng/ml TNF-α to the basolateral medium. Plates were incubated for 21 h at 37 °C, and TEER was measured again. Experiments were performed in duplicate. The results were presented as a ratio:

$$Ratio=\frac{TEER\, Treatment\, \mathrm{T}24/TEER\, Treatment\, T0}{TEER\, Control\, \mathrm{T}24/TEER\, Control\, T0}$$

Lastly, the adhesion capacity of the bacterial strains was tested following the procedure described by Turpin et al.^25^ with slight modifications. Briefly, 500 µl of a suspension of 1 × 10^5^ HT-29 MTX cells were seeded in 24-well tissue culture plates (Nunc) and cultivated to confluence. Cells were left for mucus production for a total of 21 days. The medium was changed daily. The monolayer was then infected at a multiplicity of infection (MOI) of 1:50. After 3 h of incubation at 37 °C under anaerobic conditions, the monolayers were washed three times in phosphate-buffered saline (PBS), pH 7.2. The epithelial cells were then lysed with 1% Triton X-100 (Sigma Chemical Company, St Louis, Mo.) in water. The direct samples and their respective dilutions were plated onto MRS agar plates and incubated for 48 h at 37 °C to determine the number of adhered CFUs.

### Murine model of low-grade inflammation

Male C57BL/6 mice (Janvier, Le Genest Saint Isle, France) were maintained under specific pathogen-free (SPF) conditions in the animal care facilities of the National Institute of Agricultural Research (IERP, INRA, Jouy-En-Josas, France), for a minimum of one week prior to experimentation. Mice were induced with intestinal dysfunction using DNBS as previously described^4^. Low-grade inflammation was generated by two intra-rectal injections of DNBS dissolved in EtOH (100 mg/Kg and 50 mg/Kg, respectively) under anesthesia with a 21-days recovery period in between (Fig. S1). Mice were anesthetized using an intraperitoneal (*i.p*) injection of 0.1% ketamine (Imalgene 1000, Merial, France) and 0.06% xylazine (Rompun, Bayer, France). Control mice received only the vehicle. After 30 days of the firth injection, mice were administered daily with 200 µl containing 5 × 10^9^ CFU of viable bacteria (CNCM I-3690 or 7292 and 7358 strains) or with PBS alone. A total of 80 mice were employed in this study (16 mice per group spliced in two experiments of 8 mice per group each). The experiments were realized between 2014 and 2017. All experiments were performed in accordance with European Community standards for animal care and three R rules and were approved by the relevant local committee (Comethea Ethic committee) and were performed also in compliance with the ARRIVE relevant guidelines.

Different inflammatory parameters such as macroscopic colonic score, weight loss, cytokine concentrations and myeloperoxidase (MPO) activity (a marker of the degree of infiltration by polymorphonuclear neutrophils) were measured as previously described^4^. In vivo permeability was determined using a fluorescein-conjugated dextran (FITC-dextran 3000–5000 Da (FD-4), Sigma-Aldrich) at the endpoint, as previously described^26^.

### Histological and immunohistochemical analyses

According to standard protocols, samples were fixed in 4% paraformaldehyde or Carnoy buffer, dehydrated, and embedded in paraffin^4^. Histological analyses were carried out on tissue sections blocks of 5 µm cut on a Leica RM2265 microtome and mounted on adhesive microscope slides (Superfros, ultra plus, ThermoScientific). Samples were then stained with hematoxylin–eosin Safran, periodic acid-Schiff (PAS), and Alcial blue (AB)^27,28^.

For mucin-2 (Muc2) detection, sections were deparaffinized, rehydrated, and rinsed according to standard protocols. Then, samples were incubated sequentially with the protein block (Dako, Agilent Technologies), the primary antibody (2 µl/mL, Mucin 2 rabbit polyclonal IgG, Santa Cruz Biotechnologies), and secondary antibody (2 ng/mL, Alexafluor 568 goat red anti-rabbit IgG, Invitrogen, ThermoFischer Scientific) both diluted in the Ab diluent (Dako, Agilent Technologies). The sections were then treated with trihydro-chloride trihydrate (0.5 mg/mL Hoechst 33342, Invitrogen, ThermoFischer Scientific) in PBS. Subsequently, the slides were mounted using Fluorescent Mounting Medium (Dako, Agilent Technologies).

Tissues were visualized using a high-capacity digital slide scanner (3DHISTECH Ltd.) and Panoramic and Case viewer software (3DHISTECH Ltd.).

### Transcriptome analysis

For the transcriptome analysis, 20–30 mg of colon samples were processed with a RNeasy Mini Kit (Qiagen) as previously described^19^ to extract total RNA. RNA quantity and quality were then determined using Nanodrop and lent 2100 expert Bioanalyzer, respectively. A reference design with complete dye-swap including 5 biological replicates was used to compare DNBS-challenged mice from a total of 6 Chip Agilent Sureprint G3 8 × 60K v2 Microarrays (Agilent Technologies, France). Raw data were extracted from microarray images using Agilent’s Feature Extraction software and pre-processed using the R package *agilp* obtained from Bioconductor (http://www.bioconductor.org). Probes not detected in more than 40% of the samples were excluded from the analyses. Normalization of raw intensities was done by *quantile normalization* method and normalized data were adjusted for batch effect using ComBat method.

Differential expression analysis was determined by the mean of an empirical Bayesian test. An ANOVA/Benjamini and Hochberg (multiple corrections) test was applied to identify significant genes, considering a p-value of 0.05, followed by filtering on expression level (|(FC)|> 1.25 as a cut-off)^29^. To analyze pathways and generate data displays, selected gene lists (log ratio and p-value data) were loaded into Ingenuity Pathway Analysis (IPA).

To elucidate differences in taxa abundances between the different clustering variables, an ANOVA/Tukey Kramer (post hoc) test with false discovery rate (FDR^26^) was applied, considering significant only those differences with a p-value lower than 0.05 and q-value below 0.3. All the transcriptome data have been submitted to GEO, accession number: GSE101411.

### Reverse transcription (RT) and quantitative real-time PCR (qPCR)

Reverse transcription was performed with the applied biosystems high capacity cDNA reverse transcription kit (ThermoFisher, France). For this, reverse-transcription of 1 μg of total RNAs was performed and the resulting cDNAs were then quantified using a Nanodrop (ThermoFisther Scientific Inc., France). Samples were diluted (10x), duplicated, and subjected to quantitative real-PCR (qPCR) with a Step one plus detection system (Applied). The mix reaction developed in a final volume of 25 µl, consisted of 12.5 μL of RoxSybr Master Mix blue dttp (Takyon, Eurobio, France), 1 µl of each primer (Qiagen-RT^2^ qPCR Assay), and 1 μL of diluted cDNA. Values were expressed as relative fold differences normalized to a housekeeping gene, *Gapdh,* using the 2^−ΔΔCT^method. All procedures were performed according to the manufacturers’ instructions.

### Analyses of lymphoid population

Mesenteric lymphoid nods (MLN) were gently extruded through a 50 μm-mesh Nylon cell strainer (BD) to isolate mononuclear cells. Then, cells were suspended in Dulbecco’s Modified Eagle Medium (DMEM) supplemented with 10% of FBS, 2 mM l-glutamine, 50 U/mg penicillin, and 50 µ/mg streptomycin (Lonza, Levallois-Perret, France). For flow cytometry analysis, aliquots of 10^6^–10^7^ cells per sample were labelled with anti-CD3-FICT, anti-CD4-PerCP, anti-Tbet-APC and anti-Gata3-PE according to the manufacturer’s instruction (eBioscience). The cells were analyzed using flow cytometry (Accuri, BD) and CFlow Sampler software (BD Biosciences) as described previously^30^.

### Gut microbiota analysis

Fresh fecal samples were collected at two time points (D13 and D23) and stored at − 80 °C. DNA was then obtained via mechanical lysis (Fastprep FP120 [ThermoSavant]) and phenol/chloroform-based extraction as previously described^31^. Amplification was performed using the primers V3-V4 for 16S rRNA (forward:CCTACGGGNGGCWGCAG, reverse: GACTACHVGGGTATCTAATCC). The samples were loaded into flow cells in an Illumina MiSeq. 300PE Sequencing Platform in accordance to the manufacturer’s instructions. An adaptation of the QIIME v2.0. package was used to analyze the sequence data (Caporaso et al. 2010). Briefly, read pairs were demultiplexed and trimmed (q > 20) before being merged using QIIME. Merged reads with q > 25 over a window of 15 bases, no ambiguous bases and a minimal length of 300 were retained. Taxonomic assignment was performed using the RDP classifier against the SILVA_ Silva_138 database, the results of which sequences were aggregated to different taxonomic levels. Reads with eukaryotic assignments, as well as reads with a low relative abundance up to 0.0005% in all samples were excluded from further downstream analysis. Reads with eukaryotic assignments, as well as reads with a low relative abundance up to 0.0005% in all samples were excluded from further downstream analysis. All analyses were performed using R version 3.6.0. Samples were rarefied using phyloseq library in R. R package Vegan (function *richness*) was used to calculate alpha diversity metrics (Shannon’s). Differences between changes of alpha-diversity and Bray–Curtis dissimilarities from baseline were evaluated using the Mann–Whitney tests for specific comparison, without adjustment for multiple testDESeq2 package (version 1.26.0) was used to identify genera differentially abundant within groups and between groups (~ Group + Group:Day + Mice_ID). Significant fold-change differences between doses at each time point were evaluated with the negative binomial model-based Wald test implemented in DESeq2 (FDR < 0.1). Sequences have been deposited at ENA accession number PRJEB56457.

### Statistical analysis

For the statistical data analysis, a non-parametric Kruskal–Wallis test, followed by a Dunn's Multiple Comparison as a post hoc test was employed by using GraphPad software v8 (La Jolla, CA, USA). Plots are presented as mean + /− SEM.

## Results

### Generation of putative EPS derivatives from CNCM I-3690

To isolate *L. rhamnosus* CNCM I-3690 derivatives with various EPS production levels, we used a method based on differential sedimentation during bacterial growth in semi-liquid (SL) medium^20,21,32^. Under these conditions, viscosity of SL medium restricts free movements of bacteria and sedimentation represents the principal means of displacement for bacteria which do not displays autonomous motility properties. When starting form a colony of a non-EPS-producing strain in SL medium, simultaneous growth and sedimentation result in a streaked colony^21,33^. Contrariwise, EPS-producing bacteria in these conditions are trapped in extracellular agar matrix by produced EPS and do not sediment, consequently forming round colonies. Furthermore, it is possible to observe and to select variants escaping such immobilization as they form faster sedimenting “roots” from round colonies^20^. This method has been adapted previously to select capsule polysaccharide-negative mutants^21^.

**Figure 1:**
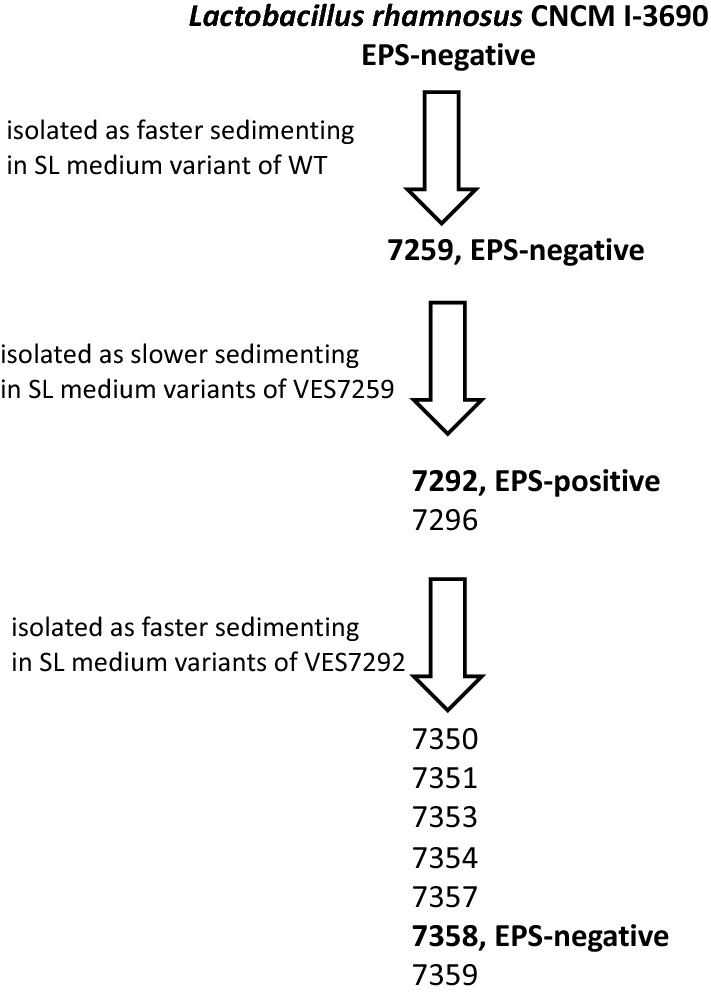
Scheme of isolation of *L. rhamnosus* CNCM I-3690 derivatives with affected EPS production.

*L. rhamnosus* CNCM I-3690 forms short chains and thus sediments relatively slowly under SL conditions. To increase the efficiency of the screening procedure, we first isolated the faster sedimenting variant named 7259 (Fig. 1). Applying the same procedure to 7259, we further isolated three slower sedimenting derivatives under SL conditions, strains 7292 and 7296 (Fig. 1). These three derivatives expressed a markedly increased ropy phenotype when grown as colonies on agar plates, a phenotype usually considered as indicative of EPS production. In addition, from derivative 7292, we isolated seven variants faster sedimenting on SL medium (strains 7350, 7351, 7353, 7354, 7357, 7358 and 7359), which on agar plates appeared as non-ropy derivatives, and therefore potentially lost their ability to secrete EPS (Fig. 1).

### The putative EPS variants of CNCM I-3690 showed altered anti-inflammatory and protective properties in vitro

**Figure 2:**
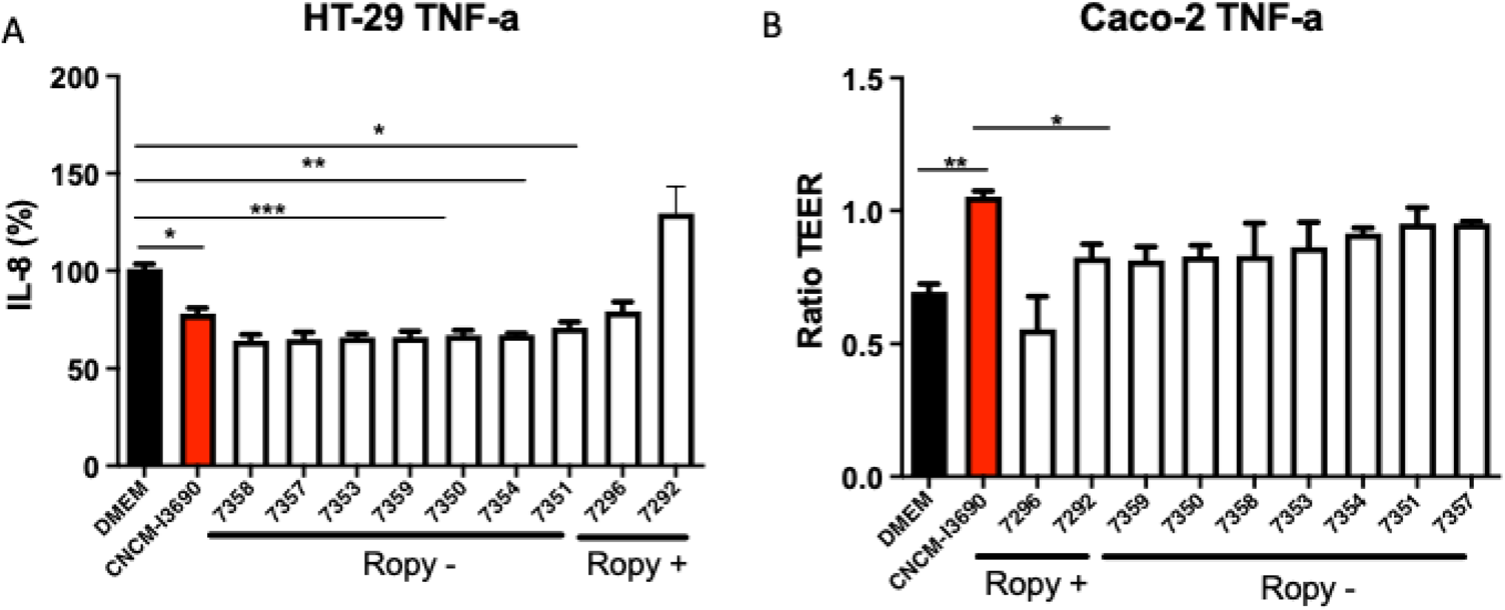
Immunomodulatory (**A**) and protective (**B**) activity of *L. rhamnosus* CNCM I-3690 and its variants. The immunomodulatory property is measured by the ability of the strains to regulate the production of interleukin 8 (IL-8) (%) by HT-29 cells stimulated with TNF-α. The protective effect is evaluated by measuring the trans-epithelial resistance in Caco-2 cells in the presence of the bacterial strains (b). Significance: * *p* < 0.05 (n = 3 × 3).

The anti-inflammatory properties of CNCM I-3690 variant strains were tested in vitro by measuring their ability to reduce the production of IL-8 by TNF-α stimulated HT-29 cells. As expected, when the WT strain CNCM I-3690 was co-incubated with the cell line, there was a statistically significant reduction in the IL-8 production (Fig. 2A). Similarly, all the non-ropy variants tested showed anti-inflammatory activity, in contrast to the ropy ones that did not show this effect. It is noteworthy the activity of the variants 7292 and 7358, the first one characterised by its ropy phenotype and the lower anti-inflammatory activity, and the second one by the opposite.

In addition, we studied the protective effect of the bacterial strains on the intestinal barrier by measuring the trans-epithelial electrical resistance (TEER) of TNF-α stimulated Caco-2 cells (Fig. 2B). As previously reported^4^, the WT CNCM I-3690 showed a significant protective effect. For the non-ropy variants a slight but not significant protective effect was observed in this test. However, the ropy ones presented a significantly lower protective ability than the wild-type.

### The variant 7292 is an over-producer EPS variant of* L. rhamnosus* CNCM I-3690 with reduced adhesion capacities

**Figure 3:**
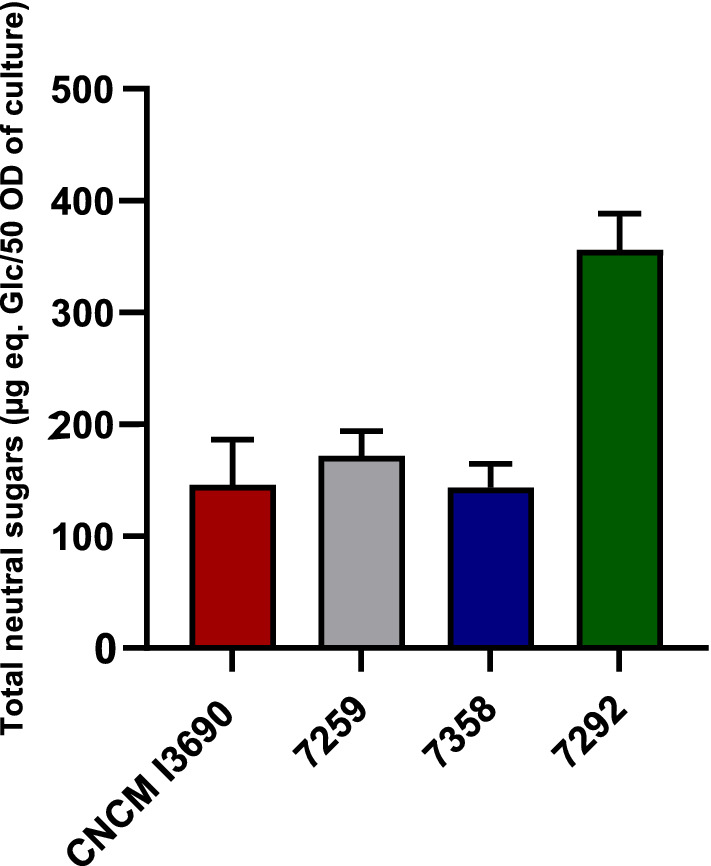
Relative EPS production of *L. rhamnosus* CNCM I-3690 and its derivatives.

Ropy phenotype is often a consequence of increased production of EPS in bacteria^34^. Since strain 7292 expressed ropy phenotype, and 7358 lost it, we performed EPS quantification experiments. For this, EPS produced by bacteria grown on filters on MRS agar plates, were isolated as previously, and the total neutral sugars in the EPS extracts were quantified. The results, presented in Fig. 3, confirm that strain 7292 indeed produces almost twofold higher amount of EPS compared to either WT or to 7259 strain. EPS production of 7358 was as low as that of WT strain. These results confirm that 7292 is an EPS-overproducing derivative of *L. rhamnosus* CNCM I-3690 and 7358 is its derivate EPS-negative variant.

**Figure 4:**
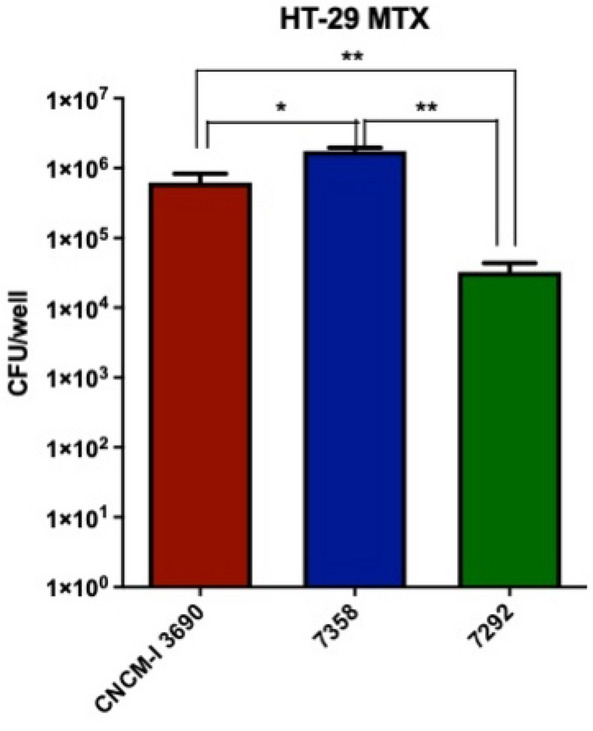
Percentage of adhesion (%) to HT-29-MTX cells of the EPS-overproducer (7292) and underproducer (7358) variants in comparation with the wild-type *L. rhamnosus* CNCM I-3690. Significance: * *p* < 0.05 (n = 3 × 3).

The variants 7358 and 7292 were then selected for further analysis and their adhesion properties were tested in the mucus producer HT-29 MTX cell line. We observed a significant reduction of the adhesion capacity of the variant 7292, whereas variant 7358 slightly increased the adhesion to the cell line compared to the WT (Fig. 4).

### Identification of mutations leading to EPS overproduction by DNA nucleotide sequence determination

We have determined DNA nucleotide sequence of WT strain CNCM I-3690, of its faster sedimenting derivative 7259, of EPS overproducing variant of 7259 (strain 7292) and of EPS-negative variant of 7292 (strain 7359). After analysis of the sequences, the mutations occurring in each strain were identified (Table S1). We found that in strains 7259, 7292 and 7358 only mutations present in coding regions were in the genes *lacE* (PTS system, lactose-specific IIB component), *lacG* (6-phospho-beta-galactosidase), *lacT* (Beta-glucoside bgl operon antiterminator), and *epsD* (Tyrosine-protein kinase). However, the *lacE*, *lacG* and *lacT* genes were incomplete in WT, and presumably inactive (Fig. S2). Therefore, we consider, that the only mutations which seems to be relevant to investigated phenotypes, the EPS production and anti-inflammatory properties, appeared to be in *epsD* gene. This gene is located in the cluster, responsible for polysaccharide synthesis and encodes regulatory tyrosine kinase, involved in regulation of EPS in *Streptococcus thermophilus* and in *S. pneumoniae*^35,36^. In the strain 7292 the mutation in *epsD* resulted in amino acid change from Ser to Leu in the position 71. We presumed that this mutation results in increased EPS production. Since strain 7358 is derivative from 7292, it had the same mutation in *epsD* and additional mutation (from Leu to Arg in position 185) in the same gene, probably resulting in inactivation of EpsD and subsequent abolishing of EPS production (Fig. S3).

Note that variant 7259 was selected as faster sedimenting derivative of WT strain, the property needed for more efficient further selection of slower sedimenting derivatives. It appeared that this strain acquired large 100 kb deletion in the terminus part of the chromosome. Deletion of this fragment is probably responsible for selected faster sedimenting phenotype due to forming shorter chains (Fig. S4). Since all derivatives of 7259, which differ in EPS production and in anti-inflammatory properties, carries this deletion, we consider that it is not relevant to mentioned phenotypes (Fig. 2).

After preliminary tests on HT-29 and Caco-2 cells (Fig. 2), ropy strain 7292 and its non-ropy derivative 7358 were chosen for further characterization.

### The EPS over-producer variant 7292 reduces the protective effects of the CNCM I-3690 wild type in a low-grade inflammation murine model

**Figure 5:**
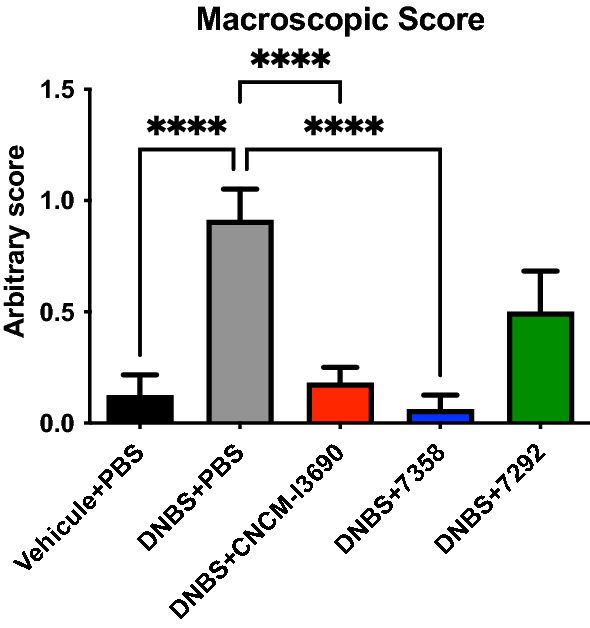
Evaluation of the macroscopic score in a dinitro-benzene sulfonic acid (DNBS) low grade colitis model after treatment with the EPS variants 7292 and 7358 in comparation with the wild-type *L. rhamnosus* CNCM I-3690. Significance: * *p* < 0.05.

**Figure 6:**
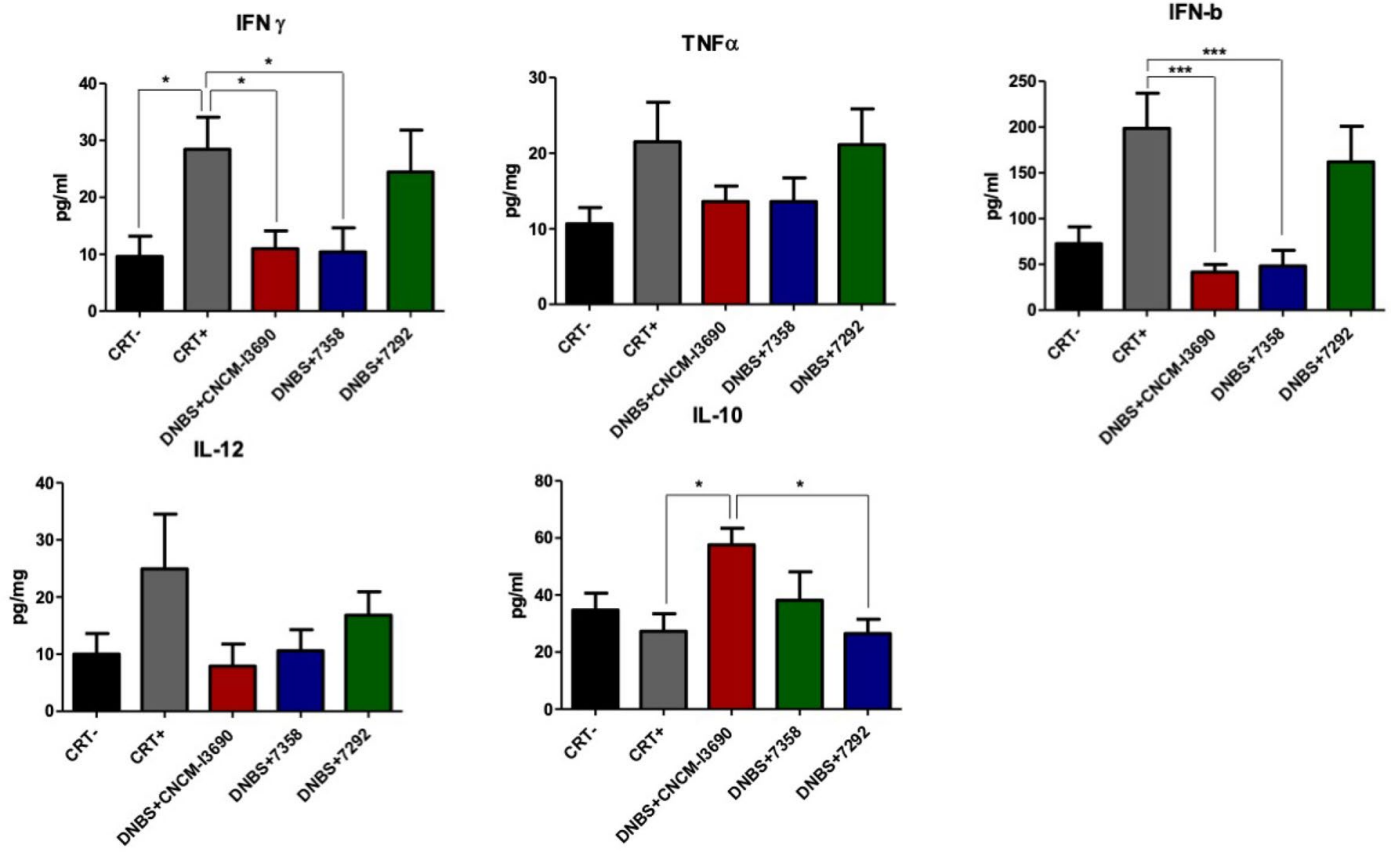
Colonic interleukin levels in a low grade dinitro-benzene sulfonic acid (DNBS) colitis model after treatment with the the EPS variants 7292 and 7358 in comparation with the wild-type *L. rhamnosus* CNCM I-3690. Significance: * *p* < 0.05.

**Figure 7:**
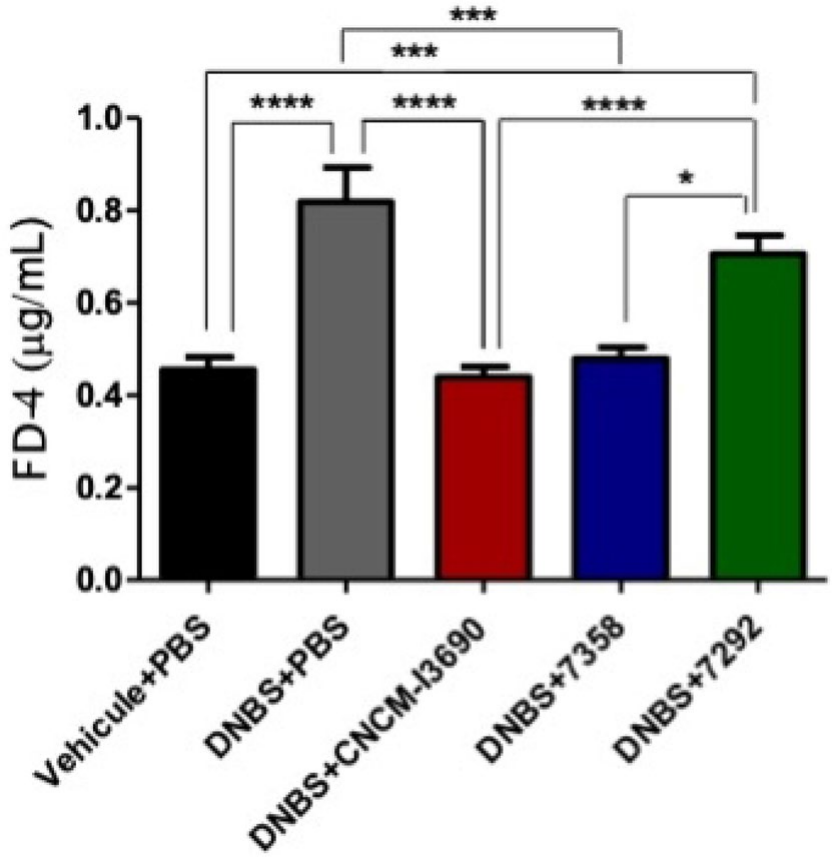
Measure of the in vivo gut permeability in a DNBS low grade murine model after treatment with the EPS variants 7292 and 7358 in comparation with the wild-type *L. rhamnosus* CNCM I-3690. Significance: * *p* < 0.05.

A murine model of chronic DNBS-induced low-grade inflammation was used to further analyse the impact of the EPS on CNCM I-3690 effects through the test of the EPS variants 7292 and 7358 (Fig. S1). Treatment with the 7358 variant improved the colonic macroscopic score, showing no statistical differences with the WT *L. rhamnosus* CNCM I-3690 (Fig. 5), while treatment with 7292 did not. Furthermore, the variant 7358, like the WT strain, reduced the levels of the cytokines and, in particular the levels of IFN-γ, and IFN-β (*p* < 0.05), and increased the level of IL-10 (Fig. 6). This effect was not observed with strain 7292 which showed similar values to the untreated group. Similarly, while FD4 permeability was increased in inflamed control and the 7292-treated mice due to DNBS challenge, it was unaltered in either the control group or in the CNCM-I 3690 and 7358-treated groups (Fig. 7).

### The EPS over-producer variant 7292 lost the capacity to improve the colonic barrier permeability by increasing mucus production and restoring the Goblet cells (GC) population

**Figure 8:**
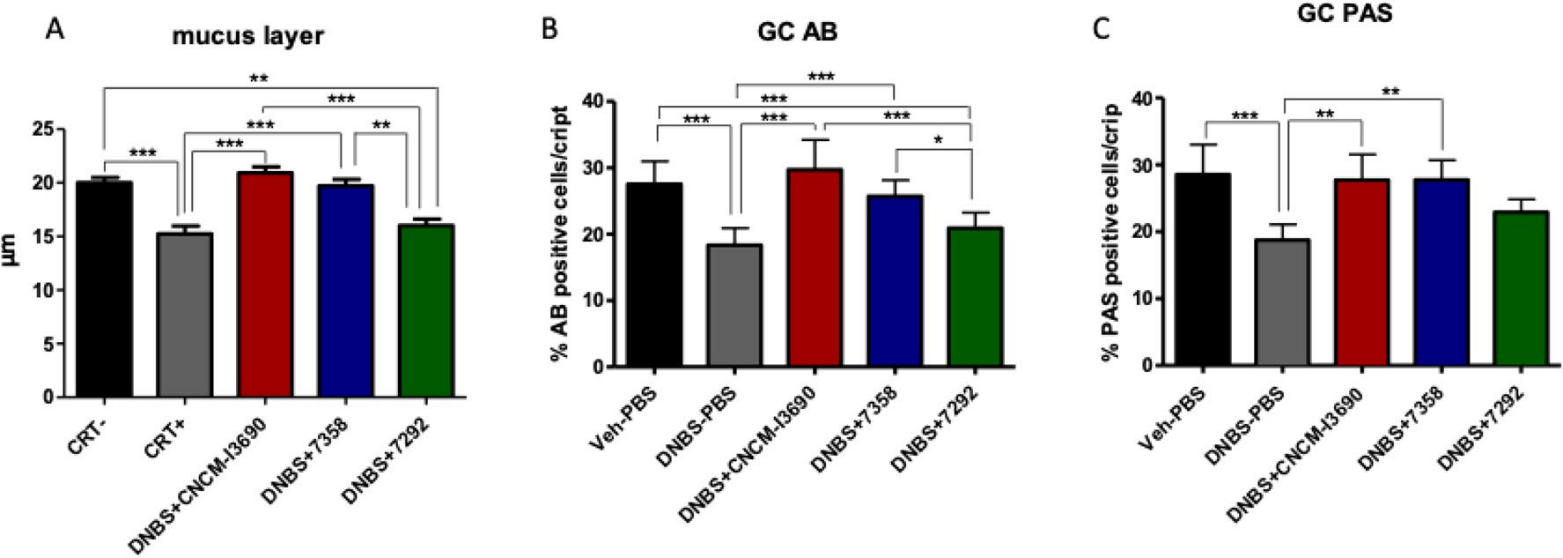
Effects of the treatment with the EPS-overproducer (7292) and underproducer (7358) variants in comparation with the wild-type *L. rhamnosus* CNCM I-3690 on the mucus layer (A), and globet cell abundance through the AB-staining (B) and PAS (C) test in the DNBS low grade murine model. Significance: * *p* < 0.05.

*L. rhamnosus* CNCM I-3690 is known to restore goblet cells (GC) populations and mucus production, both of which are altered by DNBS low-grade colitis model^4^. For this reason, we tested the ability of the variants to restore the mucus layer and mucus-producing cells. As shown in Fig. 8A, and as expected, the mucus layer thickness was significantly altered in the untreated group, which presented a thinner layer than the control, CNCM I-3690, and 7358-treated groups (*p* < 0.05). This effect could not be counteracted in the 7292-treated group which showed no significant differences with the untreated mice. The GC were stained with the Alcian blue (AB) or periodic acid-Schiff (PAS) method, which reveals acid or neutral mucopolysaccharides, respectively (Fig. 8B,C). The percentage of GC labelled either by AB or PAS was lower in the untreated group than in the CNCM I-3690 and 7358-treated groups (*p* < 0.05), which presented no significant differences among them. The 7292-treated group showed lower GC proportions, suggesting its loss of the ability to restore GC levels.

### Mesenteric lymph nodes (MLN) populations do not show significant differences among EPS variants and WT strain

**Figure 9:**
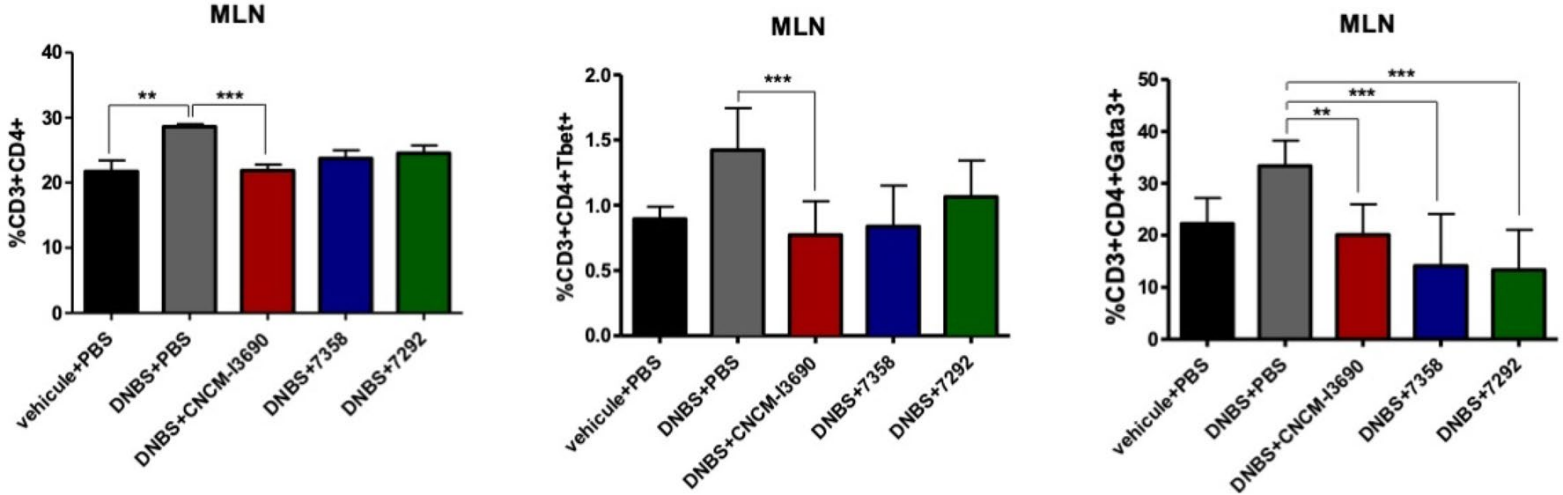
Immunological activity in MLN cells. MLN cells positive for CD3 + , CD4 + , T-bet, or GATA-3 as detected using Flow cytometry. Significance: * *p* < 0.05.

Similarly, to analyse the ability of the EPS variants to maintain the beneficial effects of the WT strain, CD3^+^/CD4^+^ T cells as well as the T-bet and GATA-3 subpopulations (Th1 and Th2 respectively) were analysed in the MLNs. As previously described^4^, values were higher in the untreated group than in the control one (*p* < 0.05) with CNCM I-3696 being able to restore the normal situation (Fig. 9). Regarding 7292 and 7358 variants, no significant differences were found when compared to CNCM I-3690 strain (Fig. 9).

### Transcriptomic analysis reveals that 7292 variant positively regulates genes related to inflammatory functions and negatively regulates protective ones

**Figure 10:**
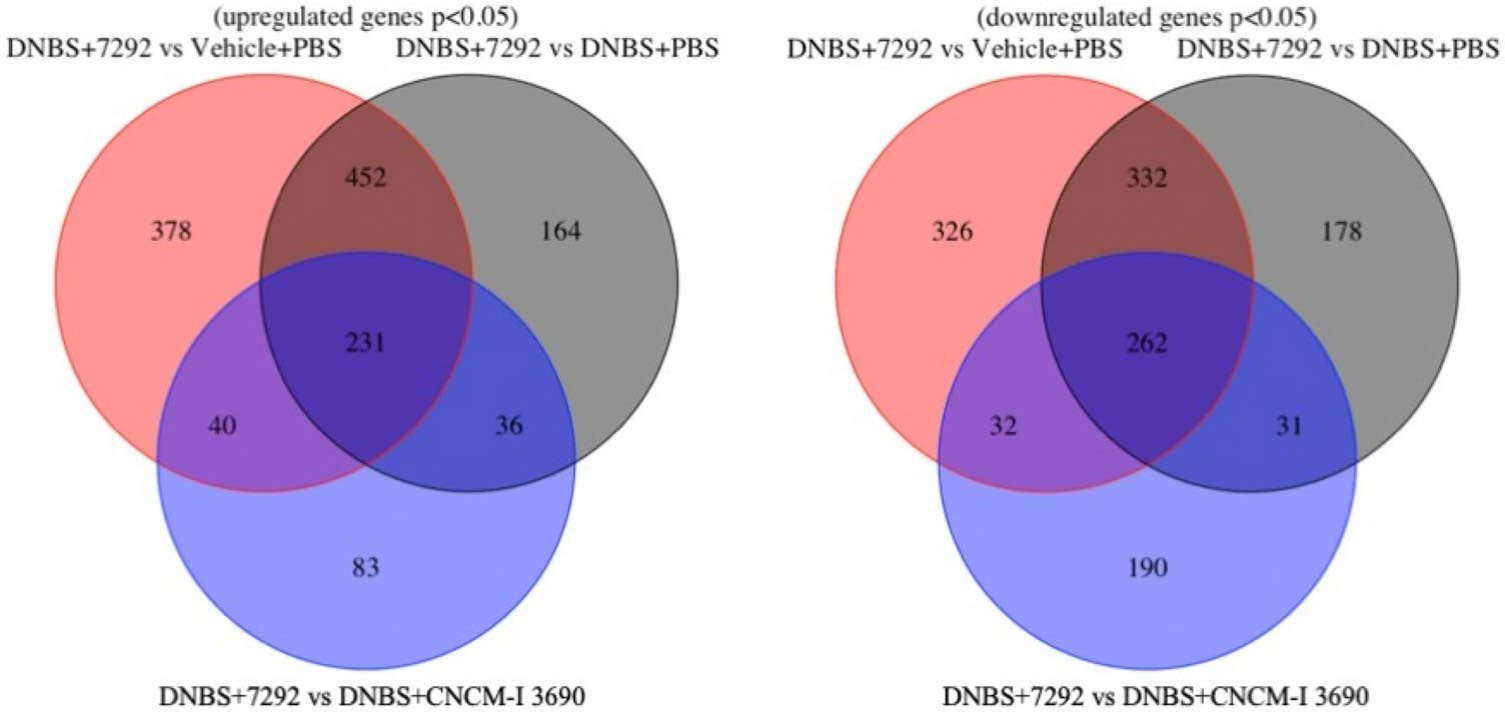
Venn diagram showing the number of unique and shared upregulated (A) and downregulated (B) genes as identified via IPA in the murine model after treatment with the variant 7292. Gene number were compared with those modulated after treatment with the wild-type *L. rhamnosus* CNCM I-3690, not-treated, and mice administered with the vehicule. Significance: * *p* < 0.05.

Transcriptomic analysis revealed the differential expression of 1686 genes (52.37% up-regulated) between the 7292-treated mice and the not treated ones, and 905 (43.09% up-regulated) between the first one and the 3690-treated mice (*p* < 0.05) (Fig. 10). IPA analysis of the specific signaling pathways modulated by the 7292 variant showed that this derivative was able to significantly upregulate (*p* < 0.05) a great number of related-immune response pathways compared to the untreated mice (Table 1).

**Table 1:**
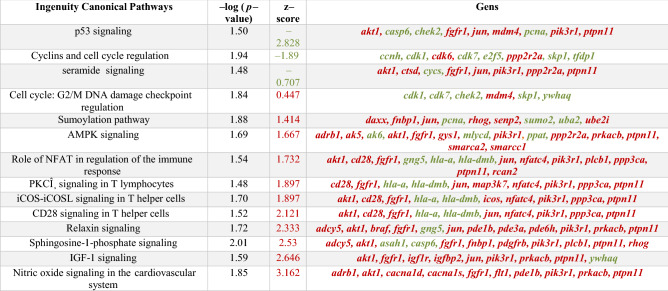
Genes modulated by the variant 7292 versus the wild-type CNCM I-3690 and the major modulated pathways modulated by the variant 7292 as identified via Ingenuity pathway analysis software.

Red color indicates upregulation, and green color downregulation.

The 7292 variant increased NF-κB, IL-6, and IL-8 signaling. There was also an increase in the macrophage derived-activity by activating different related-pathways, such as Fcγ Receptor-mediated Phagocytosis in Macrophages and Monocytes CCR5 Signaling, and production of Nitric Oxide and Reactive Oxygen Species in Macrophages, in addition to the Th1 Pathway.

The analysis also revealed an increase in the pathway involving lymphocytes, such as CTLA4 Signaling in Cytotoxic T Lymphocytes or Protein Kinase C in T lymphocytes. Besides, there was an increase in integrin signaling. Other important pathways such as ERK/MAPK signaling or Role of JAK1 and JAK3 in gc Cytokine Signaling were also activated in mice treated with the variant 7292 compared to the untreated mice.

Lastly, when compared samples 7292-treated mice with those treated with the WT strain, a down-regulation of the p-53 pathway was observed as well as up-regulation of the Nitric oxide (NO) signaling (Table S2).

### The effect of CNCM I-7292 on host is independent of gut microbiota

**Figure 11:**
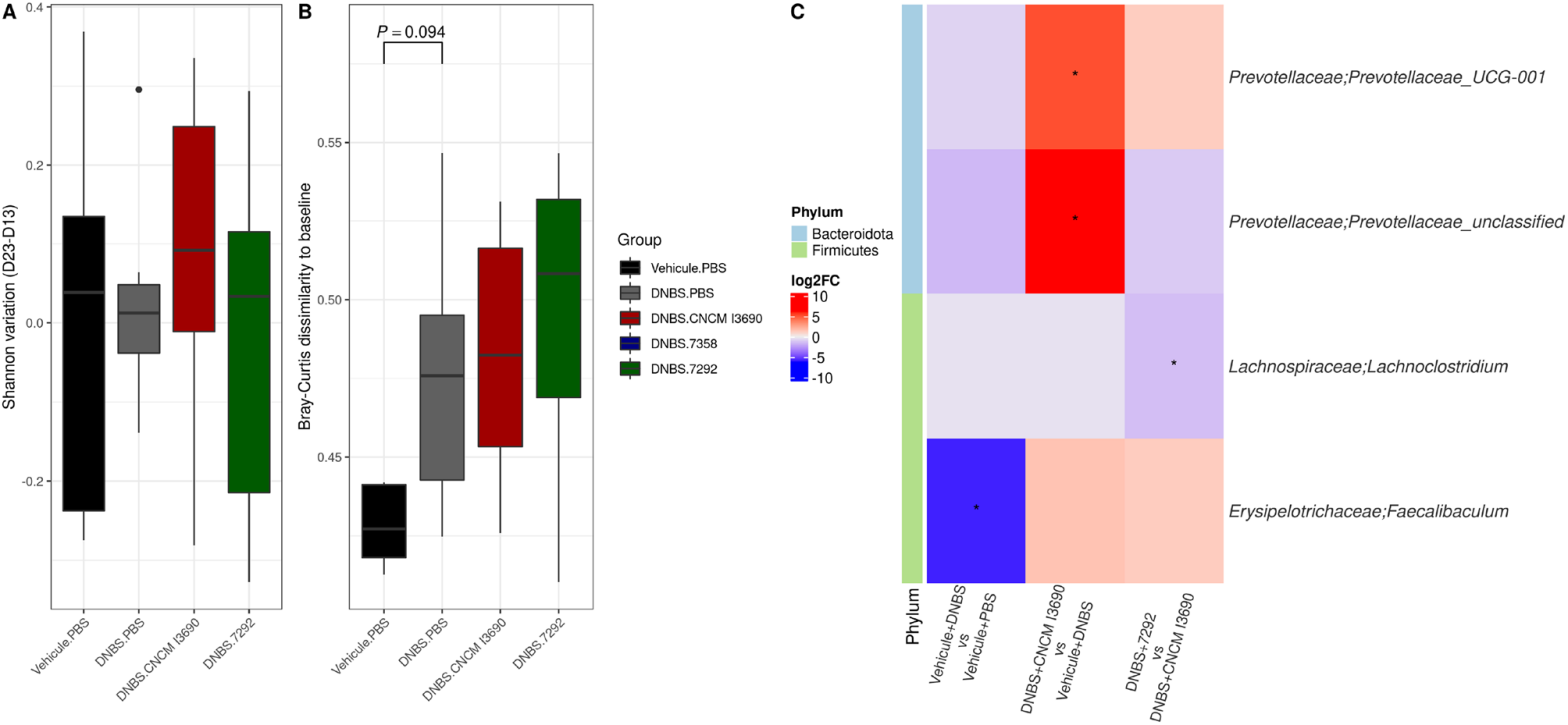
Effect of DNBS and EPS variation on gut microbiota. A) Genus-based Shannon index (Mann–Whitney test; B) Within-mice Bray–Curtis dissimilarity to D13 (Mann–Whitney test) C) Differentially abundant genera were identified using Deseq2 based and between-group effects with adjustment for D13. *FDR < 0.1. Red indicates higher abundance in the first group at D23 versus D13 compared to second group at D23 versus D13.

Finally, given the effect of the EPS over-producering variant on host, we examined whether this effect might be mediated through gut microbiota. The gut microbiota of the mice was dominated by Bacteroidota (genera within Muribaculaceae family) and Firmicutes (Fig. S5). We studied the effect of the DNBS injection, *L. rhamnosus* CNCM I3690 and 7292 derivative on the gut microbiota composition and compared specifically the effects of EPS variants 7292 to CNCM I-3690. Based on overall structure, there was no difference in alpha-diversity (genus based -Shannon index) between the analyzed groups (Fig. 11A) (Mann–Whitney, *p* > 0.05). The Bray–Curtis dissimilarity to day13 tended to be higher in mice exposed to DNBS compared to PBS (Mann–Whitney, *p* = 0.09), with no differences in groups treated with CNCM I-3690 compared to DNBS or variants 7292 compared to CNCM I-3690 (Fig. 11B). Last, differential analysis using DESeq2 analysis showed a decrease in a genus *Lachnoclostridium* in LR 7292 compared to CNCM I-3690 over time (FDR = 0.09). Taken together, these results indicate a minor effect of the 7292 on gut microbiota, suggesting most probably a direct effect on host (Fig. 11C).

## Discussion

Extracellular polysaccharides produced by probiotic microorganisms might play a prominent role in their therapeutical activities. In the present study, we evaluated the part of the production of secreted EPS in the beneficial effect on the host of the *L. rhamnosus* strain CNCM I-3690, described in previous studies as a restorer of the intestinal barrier in a murine model^4,19^. For this, we selected and analysed in vitro ten variants of this strain with altered EPS production using ropy phenotype as readout. Ropy phenotype is commonly associated with high EPS production as well as a characteristic sugar composition^22^. Subsequently, two strains with opposite phenotypes as regards ropiness were selected for further in vivo analysis. EPS quantification indicated, that 7292 produced increased amounts of EPS, whereas EPS production of 7358 was as low as WT strain. Whole genome DNA sequence analysis indicated, that 7292 acquired mutation in *epsD* gene, and 7358 acquired the second mutation in the same gene. Since *epsD* was shown to encode regulatory tyrosine kinase, involved in regulation of EPS in *S. thermophilus* and in *S. pneumoniae*^35,36^, localization the mutations in this gene is coherent with their EPS production levels.

The result of the in vitro tests showed that all the non-ropy variants conserved the anti-inflammatory effect of the WT strain. In contrast, variant 7292, characterized by its ropy phenotype, lost it. Furthermore, the ropy variant 7292 does not display the beneficial effect produced by the WT strain also in the in vivo model. In this sense, Yılmaz et al.^37^ previously reported the pro-inflammatory profile of ropy EPS produced by *Lactiplantibacillus plantarum* strains through the stimulation of IL-12 and TNF-α, in addition to limited stimulation of anti-inflammatory cytokines in HT-29 cells. In contrast, another study has shown the protective effect of a ropy EPS-producing strain *Bifidobacterium longum subsp. longum* YS108R in a DSS-induced colitis model through the maintenance of the mucosal barrier and the modulation of the gut microbiota^38^. Another example is the keystone commensal bacterium *Faecalibacterium prausnitzii*, which EPS has been described as anti-inflammatory, modulating intestinal immunity^39^, and also showing protective effect in models of chronic moderate and severe DNBS-induced colitis^40,41^.

EPS can interact with receptors located in the intestinal epithelium^42^, acting as effector molecules that elicit immune responses^43^. However, in vivo studies performed with different EPS-producing *Lactobacillus johnsonii* strains determined that mutants inactivated for EPS genes exhibited longer residence time in the animal’s gut^44,45^. Lebeer et al*.*^46^ previously demonstrated that the loss of EPS production in *L. rhamnosus* GG improved the in vitro adhesion to Caco-2 cells. However, it was also shown that the presence of EPS surrounding the WT GG strains improved in vivo performance allowing the strain to persist for longer periods because EPS protection against the antimicrobial peptide LL-37^47^. These studies support the hypothesis that the activity developed by the strain depends largely on the composition of the EPS, but also on the amount produced. Accordingly, polymers, such as EPS, could avoid the display of several bacterial surface molecules involved in adhesion and can also act as a buffer against host immune defense^43^. This is the case of pili structures that are fundamental in the beneficial effects of several probiotic and commensal bacteria^48,49^. Pili regulates host cell genes involved in cell proliferation and inflammation at colon^4,50–52^. Of note, the inactivation of the *spaF* gene in *L. rhamnosus* CNCM-I 3690 resulted in the loss of effects of the strain^4^.

Using an in vitro approach, we first observed that the EPS overproduction resulted in the loss of the anti-inflammatory effects. Similarly, mice with induced low-grade inflammation and treated with the overproducer variant did not fully recover as when treated with the WT: their colonic permeability, colonic cytokine levels, and GC populations remained altered.

Colonic transcriptome analysis revealed that the treatment with the 7292 variant changed the expression of a large number of genes. Inflammation pathways are frequently controlled by NF-κB, which is a master regulator of gene transcription^53^. The results of the transcriptomic analysis showed an increase in the NF-κB signaling in the 7292-treated mice, resulting in an exacerbated inflammation. Activation of canonical NF-κB pathway is associated with the rapid and acute production of diverse pro-inflammatory mediators, such as IL-6 or IL-8 cytokines^54^. In our study, signaling of both cytokines was increased in the 7292-treated mice with respect to the untreated mice. We previously reported the inactivation of this pathway after the administration of the WT *L. rhamnosus* CNCM I-3690^4^.

Evidence suggests that macrophages and T cell-derived cytokines play a key role in the amplification and perpetuation of the inflammatory response^55^. We observed an increase in the macrophage activity through the activation of different pathways. Among them we found Fcγ Receptor-mediated Phagocytosis, CCR5 Signaling, and production of Nitric Oxide and Reactive Oxygen Species. Besides, we also observed an increase in the Th1 Pathway. Th1 cells are essential for protecting against pathogens. They mainly produce interferon (IFN)-γ and tumor necrosis factor (TNF) that are able to activate macrophages and cytotoxic CD8 + T cells. This activation promotes the clearance of intracellular pathogens^56^. The transcriptomic results confirm those obtained in colonic cytokines analysis, as we observed an increase in the proinflammatory response through the production of IFN-γ, IFN-β, and TNF-α cytokines in mice administered with the overproducing EPS variant 7292. In contrast, the production of IL-10, an important immunoregulatory and anti-inflammatory cytokine involved in the preservation of the intestinal mucosal barrier by suppressing protein misfolding and endoplasmic reticulum stress in GC^57^ was inhibited in mice administered with 7292 with respect to the WT, whose response was high, as previously reported^4^. Therefore, it is not surprising that mice treated with the 7292 variant had significantly flower number of GC than the WT and the 7358 variant, reaching similar values that the untreated mice. GC cells are responsible for mucus production^58^, so altering their number implies a direct effect on the mucus layer, and consequently leads to permeability disfunction, as we observed in this study.

The underlying pathobiology of gut inflammation includes an increase in infiltrating gut-homing lymphocytes. Lymphocytes homing is typically a tightly regulated and stepwise process involving multiple integrins^59^. Transcriptomic analysis revealed, first, an increase in the pathway involving lymphocytes, such as CTLA4 Signaling in Cytotoxic T Lymphocytes or Protein Kinase C in T lymphocytes. Secondly, there was also an increase in integrin signaling. Lastly, other important inflammation-related pathways such as ERK/MAPK signaling or Role of JAK1 and JAK3 in gc Cytokine Signaling were also upregulated. These pathways are activated by cytokines secreted in the inflammatory response. First, there was an activation of the protein kinase (MAPK) pathway by binding to a cytokine receptor tyrosine kinase, which also activates Janus Kinase-3 (Jak-3). This signal is transferred from Jak-3 to the DNA nucleus of the cell by a chain of kinases, ultimately activating extracellular receptor kinase (ERK/MAPK)^60^. In this context, we previosuly reported higher activity of both pathways in mice treated with *L. rhamnosus* CNCM I-3690 *ΔspaF* mutant lacking pili structures, comparing to the WT strain^4^. None of the above pathways was altered in mice treated with the WT *L. rhamnosus* CNCM I-3690.

Transcriptome analysis of samples from 7292-treated mice showed a down-regulation of the p-53 pathway as well as up-regulation of the Nitric oxide (NO) signaling compared to WT. The *p-53* gene encodes for a protein responsible for cell cycle regulation, DNA repair, and apoptosis^61,62^. Overexpression of p-53 predicts the risk of colorectal neoplasia in inflammatory bowel diseases (IBD)^63^. In turn, Nitric oxide production is increased in IBD tissues. There is also evidence that the level of NO synthase enzyme (iNOS)-derived NO correlates well with disease activity in ulcerative colitis (a type of IBD)^64^.Overall, the transcriptomic analysis revealed an increase in inflammation-related signaling pathways in 7292-treated mice relative to untreated mice, thus confirming the results obtained in both in vitro and low-inflammation murine models.

Last, we evaluated the effect of the EPS over-production in host could be mediated through gut microbiota, as previous studies suggest that EPS could be a fermentable substrate for some gut microbes^65^. According to the overall structure (alpha and beta-diversity), there was no difference between groups. Differential analysis revealed minor effect of the over-expression strain suggesting that over-expressing strain exert rather a direct on host than through gut microbiota modulation.

In conclusion, in this work we show that EPS overproduction negatively affects the beneficial properties of the WT strain. We demonstrated that EPS are major effectors on the bacterial host crosstalk which effect might be species or even strain specific. The role of EPS, and its possible interference with pili functions, should be further analyzed to better decipher the gut microbiota- host crosstalk and to develop the most appropriate strategy for the use of probiotics in the treatment/prevention of intestinal inflammation.

## Supplementary Information

Supplementary Figure S1.

Supplementary Figure S2.

Supplementary Figure S3.

Supplementary Figure S4.

Supplementary Figure S5.

Supplementary Table S1.

Supplementary Table S2.

## Data Availability

The datasets used and/or analysed during the current study available from the corresponding author on reasonable request. All the transcriptome data have been submitted to GEO, accession number: GSE101411. Sequences have been deposited at ENA accession number PRJEB56457.

## References

[1] Zheng, J. *et al*. A taxonomic note on the genus *Lactobacillus*: Description of 23 novel genera, emended description of the genus *Lactobacillus* Beijerinck 1901, and union of *Lactobacillaceae* and *Leuconostocaceae*. (2020). 10.1099/ijsem.0.004107 32293557

[2] Canani RB (2016). *Lactobacillus rhamnosus* GG-supplemented formula expands butyrate-producing bacterial strains in food allergic infants. ISME J..

[3] Korpela K (2016). *Lactobacillus rhamnosus* GG intake modifies preschool children’s intestinal microbiota, alleviates penicillin-associated changes, and reduces antibiotic use. PLoS ONE.

[4] Martín R (2019). The potential probiotic *Lactobacillus rhamnosus* CNCM I-3690 strain protects the intestinal barrier by stimulating both mucus production and cytoprotective response. Sci. Rep..

[5] De Valdez GF, Torino MI, De Vuyst L, Mozzi F (2003). Food grade heteropolysaccharides. On going research and future trends on biopolymers from lactic acid bacteria. Appl. Biotechnol. Food Sci. Policy.

[6] De Vuyst L, Degeest B (1999). Heteropolysaccharides from lactic acid bacteria. FEMS Microbiol. Rev..

[7] Mozzi F (2006). Diversity of heteropolysaccharide-producing lactic acid bacterium strains and their biopolymers. Appl. Environ. Microbiol..

[8] Vaningelgem F (2004). Biodiversity of exopolysaccharides produced by *Streptococcus thermophilus* strains is reflected in their production and their molecular and functional characteristics. Appl. Environ. Microbiol..

[9] Zeidan AA (2017). Polysaccharide production by lactic acid bacteria: From genes to industrial applications. FEMS Microbiol. Rev..

[10] Sims IM (2011). Structure and functions of exopolysaccharide produced by gut commensal *Lactobacillus reuteri* 100–23. ISME J..

[11] Rehm BH (2010). Bacterial polymers: Biosynthesis, modifications and applications. Nat. Rev. Microbiol..

[12] Patel A, Lindström C, Patel A, Prajapati J, Holst O (2012). Probiotic properties of exopolysaccharide producing lactic acid bacteria isolated from vegetables and traditional Indian fermented foods. Int. J. Ferment. Foods.

[13] Azad M, Kalam A, Sarker M, Wan D (2018). Immunomodulatory effects of probiotics on cytokine profiles. BioMed Res. Int..

[14] Salazar N (2009). Exopolysaccharides produced by *Bifidobacterium longum* IPLA E44 and *Bifidobacterium animalis* subsp. lactis IPLA R1 modify the composition and metabolic activity of human faecal microbiota in pH-controlled batch cultures. Int. J. Food Microbiol..

[15] Rios-Covian D (2016). *Bacteroides fragilis* metabolises exopolysaccharides produced by bifidobacteria. BMC Microbiol..

[16] Salazar N, Gueimonde M, Hernández-Barranco AM, Ruas-Madiedo P, de Los Reyes-Gavilán CG (2008). Exopolysaccharides produced by intestinal *Bifidobacterium* strains act as fermentable substrates for human intestinal bacteria. Appl. Environ. Microbiol..

[17] Püngel D (2020). Bifidobacterium breve UCC2003 exopolysaccharide modulates the early life microbiota by acting as a potential dietary substrate. Nutrients.

[18] Grompone G (2012). Anti-inflammatory *Lactobacillus rhamnosus* CNCM I-3690 strain protects against oxidative stress and increases lifespan in *Caenorhabditis elegans*. PLoS ONE.

[19] Laval L (2015). *Lactobacillus rhamnosus* CNCM I-3690 and the commensal bacterium *Faecalibacterium prausnitzii* A2–165 exhibit similar protective effects to induced barrier hyper-permeability in mice. Gut Microbes.

[20] Mercier C, Domakova E, Tremblay J, Kulakauskas S (2000). Effects of a muramidase on a mixed bacterial community. FEMS Microbiol. Lett..

[21] Llull D, Veiga P, Tremblay J, Kulakauskas S (2005). Immobilization-based isolation of capsule-negative mutants of *Streptococcus pneumoniae*. Microbiology.

[22] Cerning J (1994). Carbon source requirements for exopolysaccharide production by *Lactobacillus casei* CG11 and partial structure analysis of the polymer. Appl. Environ. Microbiol..

[23] Dubois M, Gilles KA, Hamilton JK, Rebers PT, Smith F (1956). Colorimetric method for determination of sugars and related substances. Anal. Chem..

[24] Edgar RC (2004). MUSCLE: Multiple sequence alignment with high accuracy and high throughput. Nucleic Acids Res..

[25] Turpin W, Humblot C, Noordine M, Thomas M, Guyot J (2012). *Lactobacillaceae* and cell adhesion: Genomic and functional screening. PLoS ONE.

[26] Tambuwala MM (2010). Loss of prolyl hydroxylase-1 protects against colitis through reduced epithelial cell apoptosis and increased barrier function. Gastroenterology.

[27] Perrier C, Corthesy B (2011). Gut permeability and food allergies. Clin. Exp. Allergy.

[28] Wrzosek L (2013). *Bacteroides thetaiotaomicron* and *Faecalibacterium prausnitzii* influence the production of mucus glycans and the development of goblet cells in the colonic epithelium of a gnotobiotic model rodent. BMC Biol..

[29] Gross V (1995). Regulation of interleukin-8 production in a human colon epithelial cell line (HT-29). Gastroenterology.

[30] Martín R (2016). *Bifidobacterium animalis* ssp. lactis CNCM-I2494 restores gut barrier permeability in chronically low-grade inflamed mice. Front. Microbiol..

[31] Martín R (2017). Using murine colitis models to analyze probiotics–host interactions. FEMS Microbiol. Rev..

[32] Chapot-Chartier M (2010). Cell surface of *Lactococcus lactis* is covered by a protective polysaccharide pellicle. J. Biol. Chem..

[33] Ibrahim M (2004). Immobilisation des lactocoques. Lait.

[34] Del Piano M (2014). Assessment of the capability of a gelling complex made of tara gum and the exopolysaccharides produced by the microorganism *Streptococcus thermophilus* ST10 to prospectively restore the gut physiological barrier: A pilot study. J. Clin. Gastroenterol..

[35] Morona JK, Paton JC, Miller DC, Morona R (2000). Tyrosine phosphorylation of CpsD negatively regulates capsular polysaccharide biosynthesis in *Streptococcus pneumoniae*. Mol. Microbiol..

[36] Minic, Z. *et al*. Control of EpsE, the phosphoglycosyltransferase initiating exopolysaccharide synthesis in Streptococcus thermophilus, by EpsD tyrosine kinase. *Control of EpsE, the phosphoglycosyltransferase initiating exopolysaccharide synthesis in Streptococcus thermophilus, by EpsD tyrosine kinase* (2007). 10.1128/JB.01122-06 PMC1797369 16980450

[37] Yılmaz T, Şimşek Ö (2020). Potential health benefits of ropy exopolysaccharides produced by *Lactobacillus plantarum*. Molecules.

[38] Yan S (2019). A ropy exopolysaccharide producing strain *Bifidobacterium longum* subsp. longum YS108R alleviates DSS-induced colitis by maintenance of the mucosal barrier and gut microbiota modulation. Food Funct..

[39] Rossi O (2015). *Faecalibacterium prausnitzii* strain HTF-F and its extracellular polymeric matrix attenuate clinical parameters in DSS-induced colitis. PLoS ONE.

[40] Martín R (2015). *Faecalibacterium prausnitzii* prevents physiological damages in a chronic low-grade inflammation murine model. BMC Microbiol..

[41] Martín R (2014). The commensal bacterium *Faecalibacterium prausnitzii* is protective in DNBS-induced chronic moderate and severe colitis models. Inflamm. Bowel Dis..

[42] Lebeer S, Vanderleyden J, De Keersmaecker SC (2010). Host interactions of probiotic bacterial surface molecules: Comparison with commensals and pathogens. Nat. Rev. Microbiol..

[43] Hidalgo-Cantabrana C (2012). Immune modulation capability of exopolysaccharides synthesised by lactic acid bacteria and bifidobacteria. Probiotics Antimicrob. Proteins.

[44] Horn N (2013). Spontaneous mutation reveals influence of exopolysaccharide on *Lactobacillus johnsonii* surface characteristics. PLoS ONE.

[45] Denou E (2008). Identification of genes associated with the long-gut-persistence phenotype of the probiotic *Lactobacillus johnsonii* strain NCC533 using a combination of genomics and transcriptome analysis. J. Bacteriol..

[46] Lebeer S (2009). Identification of a gene cluster for the biosynthesis of a long, galactose-rich exopolysaccharide in *Lactobacillus rhamnosus* GG and functional analysis of the priming glycosyltransferase. Appl. Environ. Microbiol..

[47] Lebeer S, Claes IJ, Verhoeven TL, Vanderleyden J, De Keersmaecker SC (2011). Exopolysaccharides of *Lactobacillus rhamnosus* GG form a protective shield against innate immune factors in the intestine. Microb. Biotechnol..

[48] Ottman N (2017). Pili-like proteins of *Akkermansia muciniphila* modulate host immune responses and gut barrier function. PLoS ONE.

[49] Segers ME, Lebeer S (2014). Towards a better understanding of *Lactobacillus rhamnosus* GG-host interactions. Microb. Cell Fact..

[50] Waetzig GH, Schreiber S (2003). Mitogen-activated protein kinases in chronic intestinal inflammation—Targeting ancient pathways to treat modern diseases. Aliment. Pharmacol. Ther..

[51] New DC, Wong YH (2007). Molecular mechanisms mediating the G protein-coupled receptor regulation of cell cycle progression. J. Mol. Signal..

[52] Piomelli D (1993). Arachidonic acid in cell signaling. Curr. Opin. Cell Biol..

[53] Liu T, Zhang L, Joo D, Sun S (2017). NF-κB signaling in inflammation. Signal Transduct. Target. Ther..

[54] McDaniel DK, Eden K, Ringel VM, Allen IC (2016). Emerging roles for noncanonical NF-κB signaling in the modulation of inflammatory bowel disease pathobiology. Inflamm. Bowel Dis..

[55] Sartor RB (1994). Cytokines in intestinal inflammation: Pathophysiological and clinical considerations. Gastroenterology.

[56] Manetti R (1993). Natural killer cell stimulatory factor (interleukin 12 [IL-12]) induces T helper type 1 (Th1)-specific immune responses and inhibits the development of IL-4-producing Th cells. J. Exp. Med..

[57] Hasnain SZ (2013). IL-10 promotes production of intestinal mucus by suppressing protein misfolding and endoplasmic reticulum stress in goblet cells. Gastroenterology.

[58] Kim JJ, Khan WI (2013). Goblet cells and mucins: Role in innate defense in enteric infections. Pathogens.

[59] Dotan I (2020). The role of integrins in the pathogenesis of inflammatory bowel disease: Approved and investigational anti-integrin therapies. Med. Res. Rev..

[60] Setia S, Nehru B, Sanyal SN (2014). Upregulation of MAPK/Erk and PI3K/Akt pathways in ulcerative colitis-associated colon cancer. Biomed. Pharmacother..

[61] Soussi T (2005). The p53 pathway and human cancer. J. Br. Surg..

[62] Steele R, Thompson AM, Hall PA, Lane DP (1998). The p53 tumour suppressor gene. J. Br. Surg..

[63] Horvath B (2015). Overexpression of p53 predicts colorectal neoplasia risk in patients with inflammatory bowel disease and mucosa changes indefinite for dysplasia. Gastroenterol. Rep..

[64] Cross RK, Wilson KT (2003). Nitric oxide in inflammatory bowel disease. Inflamm. Bowel Dis..

[65] Everard A (2013). Cross-talk between *Akkermansia muciniphila* and intestinal epithelium controls diet-induced obesity. Proc. Natl. Acad. Sci..

