# Supplementary figures and images for: Over-production of exopolysaccharide by Lacticaseibacillus rhamnosus CNCM I-3690 strain cutbacks its beneficial effect on the host
Source: Sci Rep. 2023 Apr 14;13:6114. doi: 10.1038/s41598-023-32116-3 (PMC10104810; doi:10.1038/s41598-023-32116-3)

FIGURE S1

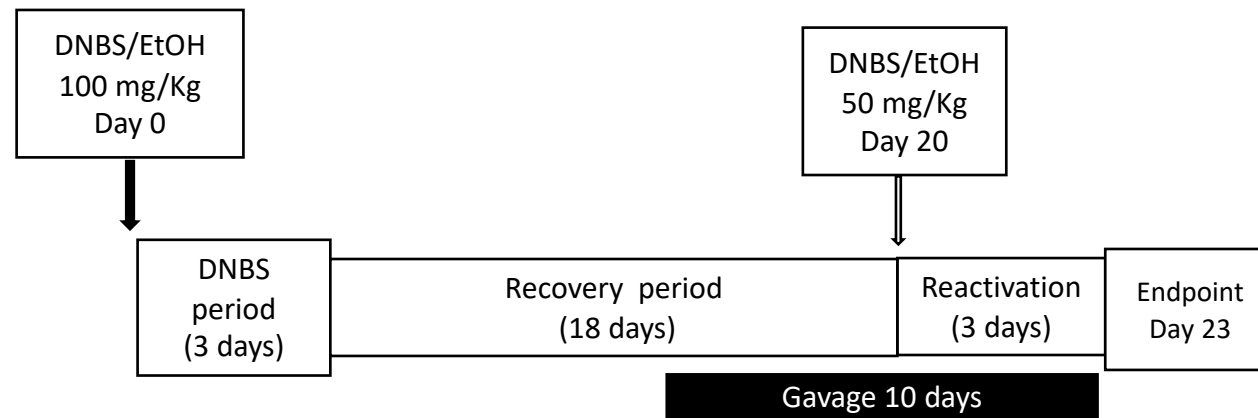

**Fig. S1. Experimental protocol for the generation of the DNBS low grade murine model.**

Supplement: Supplementary file 1 — Supplementary Figure S1. [file 41598_2023_32116_MOESM1_ESM.pdf]

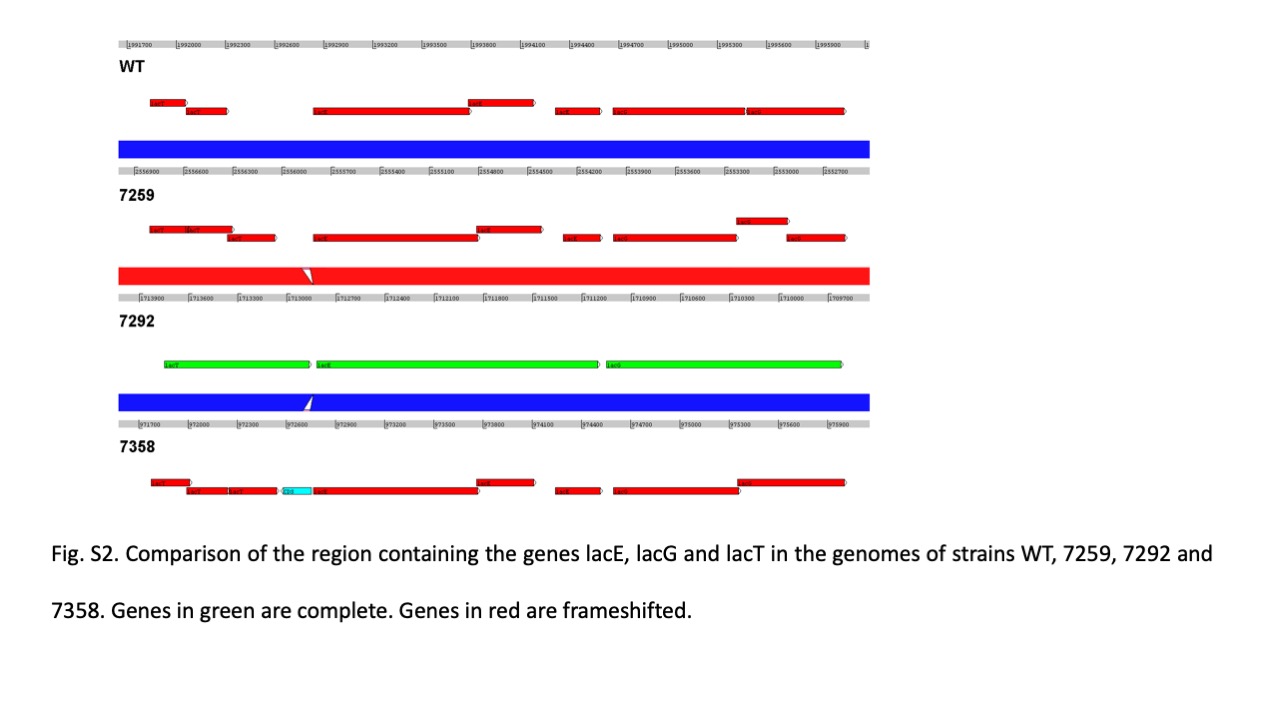

Supplement: Supplementary file 2 — Supplementary Figure S2. [file 41598_2023_32116_MOESM2_ESM.jpg]

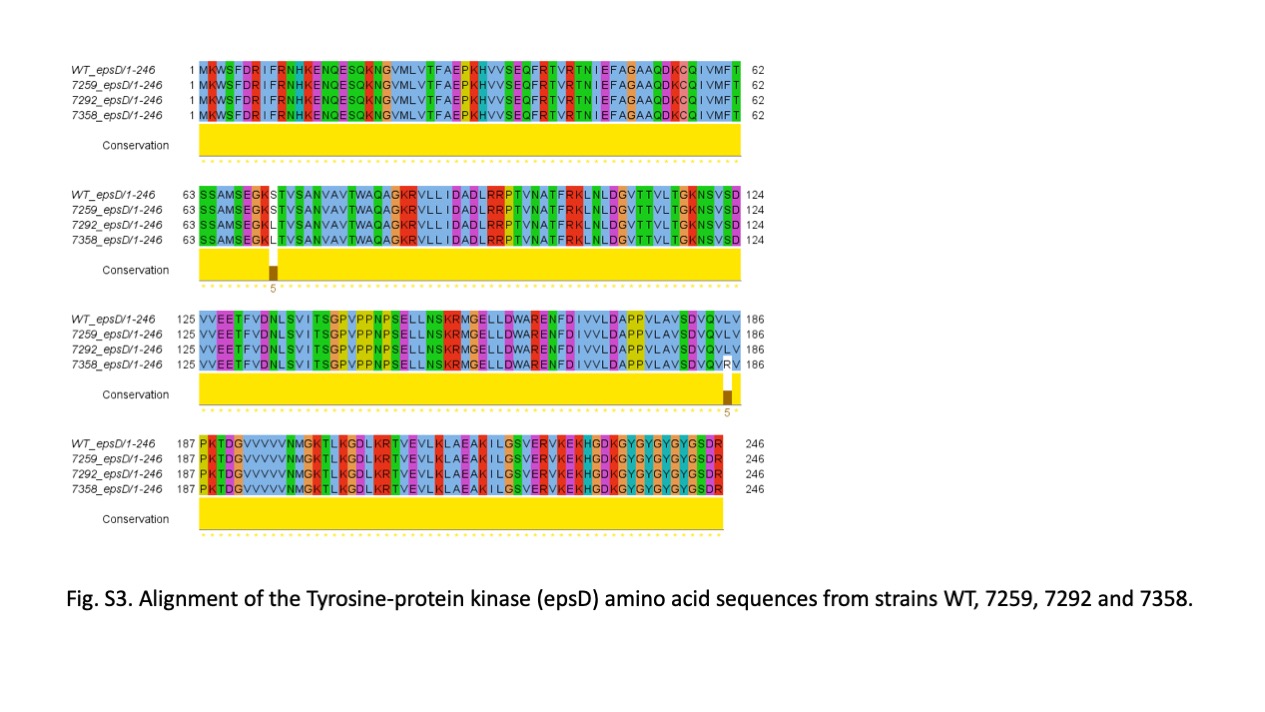

Supplement: Supplementary file 3 — Supplementary Figure S3. [file 41598_2023_32116_MOESM3_ESM.jpg]

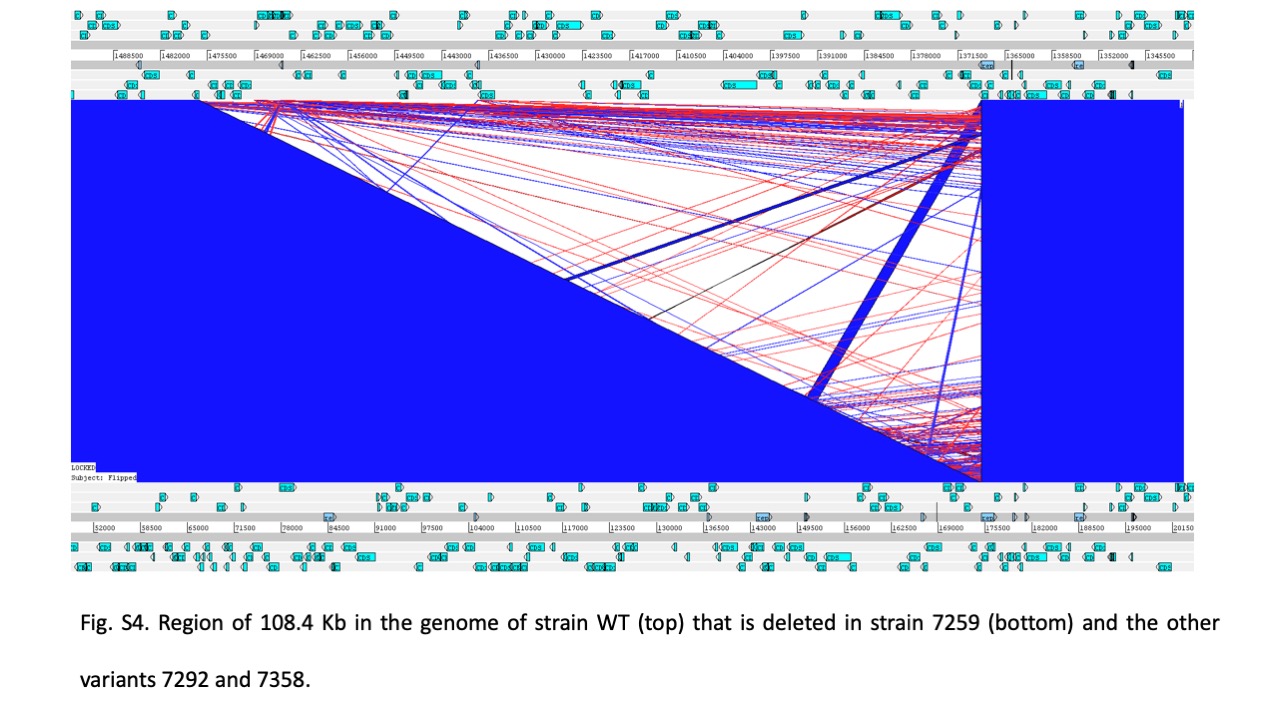

Supplement: Supplementary file 4 — Supplementary Figure S4. [file 41598_2023_32116_MOESM4_ESM.jpg]

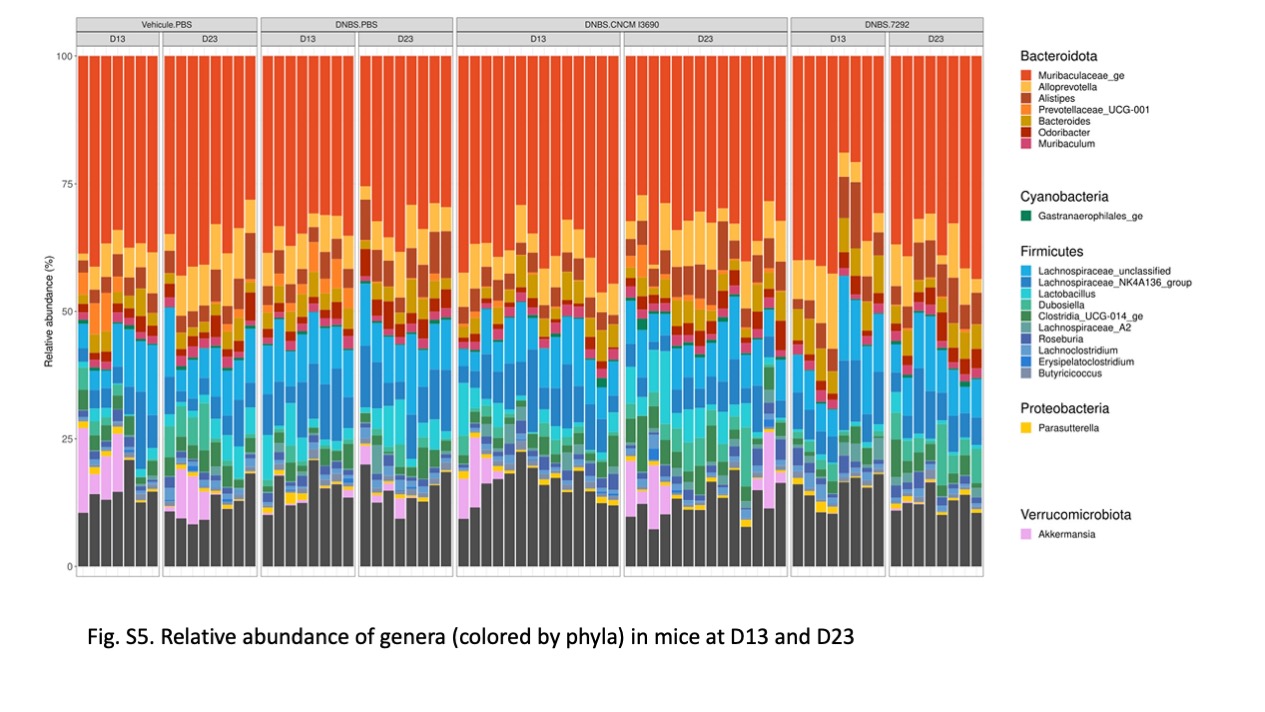

Supplement: Supplementary file 5 — Supplementary Figure S5. [file 41598_2023_32116_MOESM5_ESM.jpg]
